# Shiga Toxin–producing *Escherichia coli* Serotype O78:H^–^ in Family, Finland, 2009

**DOI:** 10.3201/eid1804.111310

**Published:** 2012-04

**Authors:** Taru Lienemann, Eeva Salo, Ruska Rimhanen-Finne, Kai Rönnholm, Mari Taimisto, Jari J. Hirvonen, Eveliina Tarkka, Markku Kuusi, Anja Siitonen

**Affiliations:** National Institute for Health and Welfare, Helsinki, Finland (T. Lienemann, R. Rimhanen-Finne, A. Siitonen);; University of Helsinki, Helsinki (E. Salo, K. Rönnholm, E. Tarkka);; Vaasa Central Hospital, Vaasa, Finland (M. Taimisto, J.J. Hirvonen)

**Keywords:** Hemolytic uremic syndrome (HUS), cross-contamination, invasive Shiga toxin–producing E. coli, STEC, stx1c, bacteremia, bacteria, zoonoses

## Abstract

Shiga toxin–producing *Escherichia coli* (STEC) is a pathogen that causes gastroenteritis and bloody diarrhea but can lead to severe disease, such as hemolytic uremic syndrome (HUS). STEC serotype O78:H^–^ is rare among humans, and infections are often asymptomatic. We detected a sorbitol-fermenting STEC O78:H^–^*stx_1c_*:*hlyA* in blood and fecal samples of a 2-week-old boy who had bacteremia and HUS and in fecal samples of his asymptomatic family members. The phenotypic and genotypic characteristics and the virulence properties of this invasive STEC were investigated. Our findings demonstrate that contrary to earlier suggestions, STEC under certain conditions can invade the human bloodstream. Moreover, this study highlights the need to implement appropriate diagnostic methods for identifying the whole spectrum of STEC strains associated with HUS.

## Abstract 2

STEC carrying *stx_1c_* and *hlyA* genes can invade the human bloodstream.

Diarrheagenic *Escherichia coli* strains, particularly Shiga toxin–producing *E. coli* (STEC), are foodborne and waterborne pathogens that cause a wide spectrum of symptoms, ranging from mild gastroenteritis to severe diseases such as hemorrhagic colitis, thrombotic thrombocytopenic purpura, and hemolytic uremic syndrome (HUS) (*1*). STEC has been characterized as a moderately invasive enteric pathogen because it is unable to invade the host cell cytoplasm but secretes phage-encoded Shiga toxin (Stx) that activates the signal pathway, leading to cell death and disease. Several reports have demonstrated the ability of STEC strains to invade epithelial cells in vitro, although in small numbers (*2**,**3*), but no reports of invasion in vivo have been published.

Stx plays a major role in intense inflammatory response and may explain the ability of STEC strains to cause HUS. The *stx* genes are located in a bacteriophage integrated into the bacterial genome, and the production of Stx is linked with the replication cycle of the phage (*4*). Stx has 2 major subfamilies: Stx1 and Stx2. Those producing variants Stx2a, Stx2c, and Stx2d_activable_ have been associated with more severe illness and HUS, whereas the other variants were often associated with uncomplicated diarrhea and asymptomatic infections (*5*). The colonization mechanism for the cell invasion is not yet fully understood, but the bacterium is known to attach firmly to the epithelial cells through an outer membrane protein called intimin. This protein is encoded by the gene *eae* on a pathogenicity island called the locus of enterocyte effacement, and the bacterial fimbriae are presumed to be involved in the process (*6*).

HUS is characterized by acute onset of microangiopathic hemolytic anemia, renal injury, and low platelet count (*7*). It is primarily a disease of infancy and early childhood because infants and young children are more vulnerable than adults, even for low Stx concentrations; however, humans of all ages can be affected. The reported STEC infections, especially with a linkage to HUS, have been frequently caused by strains of the sorbitol-negative serotype O157:H7 (*8*). However, some sorbitol-positive strains of non–O157 STEC serotypes also cause a similar spectrum of signs and symptoms (*9*).

Infections with STEC of serotype O78:H^–^ are rare among humans and often linked with asymptomatic infections. We describe a family cluster caused by STEC serotype O78:H^–^ associated with neonatal bacteremia and diarrheal (D+) HUS.

## Methods

### Case Report and Clinical Sampling

The patient, a boy born on October 3, 2009, was the third child of healthy parents. He was breast-fed and healthy. At 2 weeks of age, he became irritable, started feeding poorly, and produced large volumes of watery feces with some blood. At 17 days of age, he was taken to the Vaasa Central Hospital (Vaasa, Finland) for medical care. Blood was collected in a One BacT/Alert pediatric blood culture bottle (bioMérieux, Marcy l’Etoile, France) and incubated in the BacT/Alert automated culturing system at the clinical microbiological laboratory in Vaasa. The blood culture showed a gram-negative rod, which was identified as *E. coli*. Results of a test for the O157 antigen were negative. Because the neonate was severely ill, he was referred to the University Hospital in Pirkanmaa Hospital district, and the s *E. coli* train isolated from his blood was forwarded to the Helsinki University Hospital Laboratory, where the invasive strain from fecal specimens of the neonate and all 4 asymptomatic family members—the mother (31 years of age), father (32 years), sister (3 years), and brother (2 years)—was confirmed by detection of Stx by using the Premier EHEC EIA-test (Meridian Bioscience, Inc., Cincinnati, OH, USA). All the STEC isolates were then sent to the Bacteriology Unit (BU) of the National Institute for Health and Welfare (THL, Helsinki, Finland) for verification and more accurate phenotyping and genotyping. Fecal sampling continued until 3 consecutive STEC-negative results were obtained.

Laboratory examination indicated that the neonate had elevated levels of C-reactive protein (261 g/L [reference <3 mg/L]) and serum creatinine (246 µmol/L [reference 10–56 μmol/L). Later laboratory investigations of the neonate indicated metabolic acidosis, hyponatremia (118 mmol/L [reference 137–145 mmol/L]), and hyperkalemia (8.9 mmol/L [reference 3.3–5.2 mmol/L]). In addition, ultrasound showed enlarged kidneys. The neonate was given intravenous fluids and ceftriaxone in response to presumed sepsis. On October 27, the neonate was referred to the Department of Pediatric Nephrology and Transplantation at the University Hospital for Children and Adolescents in Helsinki for peritoneal dialysis. At admission, the neonate had a history of bloody diarrhea, STEC sepsis, thrombocytopenia, hemolytic anemia (plasma concentrations of lactate hydrogenase and hemoglobin were elevated) with fragmented erythrocytes in peripheral blood and acute uremia. Thus, D+ HUS was diagnosed. A kidney biopsy was not performed.

The neonate had low blood pressure but was anuric and overhydrated. Thus, continuous veno-venous hemodiafiltration with the support of an adrenalin infusion was started. As soon as hemodynamic and clinical conditions improved and the neonate stayed anuric, continuous veno-venous hemodiafiltration was switched to hemodialysis treatment. Because of continuously high C-reactive protein values and signs of abscess in the left kidney, a left-sided nephrectomy was performed on October 28. Histologic investigation of the kidney showed large areas of kidney necrosis, foci of abscesses, and chronic inflammation. The glomeruli of the kidneys were totally destroyed, and no typical early changes of thrombotic microangiopathy changes in HUS were seen. Peritoneal dialysis was continued because of the end-stage kidney disease but was unsuccessful because of continuous problems with hernias (2 inguinal hernia operations) and left-side pleural fluid. Thus, hemodialysis was continued, and the child was discharged on January 19, 2010, in good condition. hemodialysis treatment was performed 3×/week until April 12, 2010, when peritoneal dialysis started again in the University Hospital in Helsinki. The child had regular follow up in the outpatient clinic every 1–2 weeks in the Vaasa Central Hospital and University Hospital and every 3 months in the ward in the Hospital for Children and Adolescents in Helsinki. His nutrition was evaluated at least 1×/month by a pediatric renal nutritionist. The child’s neurologic development was normal.

His father was a suitable donor, and a kidney transplantation was performed in April 2011. The operation and posttransplantation period went without complications. After the transplantation, triple immunosuppression (cyclosporine, azatioprine, and methylprednisolone) was used, and no acute rejection episodes occurred. At discharge, the glomerular filtration rate was 91 mL/min. Through the most recent follow-up, kidney function has been stable.

### Phenotyping

Biochemical identification of the STEC strains was conducted by using an API 20E test strip (bioMérieux, Marcy l’Etoile, France). The ability to ferment sorbitol was additionally investigated on sorbitol-McConkey agar. By using the agar diffusion method with Müller-Hinton agar, susceptibility was tested for the following 12 antimicrobial agents: ampicillin, chloramphenicol, streptomycin, sulfonamide, tetracycline, ciprofloxacin, trimethoprim, gentamicin, nalidixic acid, cefotaxime, mecillinam, and imipenem (*10*). The production of the Stx1 and Stx2 was investigated by using a reversed passive latex agglutination kit VTEC-RPLA (Oxoid, Basingstoke, UK). Because all 6 strains were O nontypeable by the antiserum available at BU/THL, they were sent to the Statens Serum Institute (Copenhagen, Denmark) for further serotyping. H-typing was performed at BU/THL. Strains that were not able to migrate through a Graigie tube with semisolid agar were defined as nonmotile (H^–^) (*11*).

### Virulence Genes Detection by 16-Plex PCR

Sixteen-plex PCR was used to detect the genes *uidA, pic, bfpB*, *invE*, *hlyA*, *elt*, *ent*, *escV*, *eaeA*, *ipaH*, *aggR*, *stx_1_*, *stx_2_*, *estIa*, *estIb*, and *astA* by using the primers and PCR conditions as described (*12*). The following control strains were used: RH 4283 (E 2348/69 [(*13*]) for enteropathogenic *E. coli*, RH 4266 (ATCC 35401) for enterotoxigenic *E. coli*, RH 4270 (ATCC 43895) for STEC, RH 6647 (145–46–215, Statens Serum Institute) for enteroinvasive *E. coli*, IH 56822 (patient isolate [*14*]) for enteroaggregative *E. coli*, and RH 6715 (ATCC25922) for *E. coli* negative control.

### Pulsed-field Gel Electrophoresis

Pulsed-field gel electrophoresis using *XbaI* as restriction enzyme was performed according to the PulseNet USA protocol for *E. coli* O157:H7 (*15*). The clonal similarity index of the isolates was calculated by using unweighted pair group method with arithmetic mean clustering with the BioNumerics software version 5.10 (Applied Maths, Kortrijk, Belgium).

### Sequencing

For sequencing, the whole 1,470-bp fragment of the *stx_1_* gene (including the −10 and −35 promoter regions) from the 6 STEC strains, isolated from blood and fecal samples from the patient and fecal samples from his asymptomatic family members were amplified by using PCR primers as described (*16*). The PCR products were purified by using a MONTAGE centrifugal filter device kit (Millipore, Billerica, MA, USA). Approximately 10 ng of the PCR product was forwarded to the FIMM Technology Center sequencing laboratory (Helsinki, Finland) for sequencing by using the forward primer (5′-TCGCATGAGATCTGACC-3′) and the ABI3730xl sequencer (Applied Biosystems, Foster City, CA, USA). The sequences were analyzed by using the Bioedit program (www.mbio.ncsu.edu/BioEdit/BioEdit.html) and the homology searches by using the online National Center for Biotechnology Information GeneBlast tool (www.ncbi.nlm.nih.gov/BLAST/).

## Results

The 6 strains isolated from the blood and fecal samples of the neonate and from the fecal samples of his asymptomatic parents and 2 siblings showed a sorbitol-fermenting STEC serotype O78:H^–^ that carried the virulence genes *stx_1_* and *hlyA* (Table). The strains produced Stx1 at a titer that varied from 16 to 32. All strains were susceptible to the 12 antimicrobial drugs tested. In addition, the strains were indistinguishable in the PFGE analysis (Figure). The *stx1c* sequences obtained in this study were compared by using BLAST alignment with previously sequenced *stx1c* (AJ312232), *stx1d* (AY170851), and *stx1* (M19473) *stx1* subtypes. The strains in this study were found to be identical to the sequences within the hypervariable gene region of subtype *stx1c* (AJ312232, in positions 570–598 and 600–627). In these areas, the sequence of the subtype *stx_1c_* can be distinguished from the subtypes *stx_1_*, and *stx_1d_* (M19473 and AY170851). The sequences have been stored and gene accession codes obtained from the EMBL nucleotide sequence databank (Table).

**Table:** Characteristics of family members with Shiga toxin–producing *Escherichia coli* O78:H^–^:*stx1c*:*hlyA*, Finland, 2009

Family member	Strain characteristics, n = 6				
	Strain no.	Origin	Virulence factor	Gene accession no.	Shiga toxin titer
Neonate	FE94076 and FE94084	Blood and feces	*stx1c, hlyA*	FR875155 and FR875151	16
Brother	FE94098	Feces	*stx1c, hlyA*	FR875153	16
Sister	FE94195	Feces	*stx1c, hlyA*	FR875154	32
Mother	FE94097	Feces	*stx1c, hlyA*	FR875152	16
Father	FE94099	Feces	*stx1c, hlyA*	FR875150	32

**Figure:**
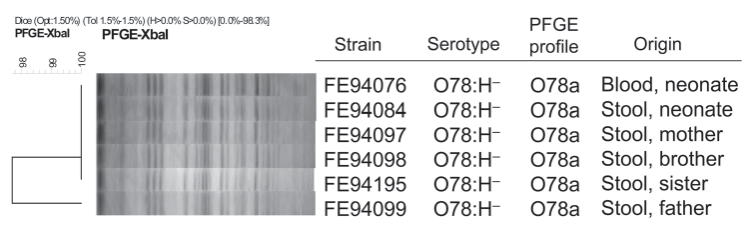
Cluster analysis of *Xba*I pulsed-field gel electrophoresis (PFGE) patterns of Shiga toxin–producing *Escherichia coli* O78:H^–^ strains isolated from blood and feces of a neonate and from feces of his asymptomatic family members, Finland, 2009. Scale bar indicates genotypic similarity of the 6 strains.

## Discussion

We found clinical evidence for the STEC O78:H^–^ infection leading to bacteremia and D+ HUS. The main cause for uremia in neonates with urosepsis is acute tubular necrosis. However, in this case, the signs fulfilled the criteria for D+ HUS and was associated with a family cluster of STEC O78:H^–^. D+ HUS–causing STEC strains generally are not found in blood; nondiarrheal HUS-causing STEC strains have been reported to cause bacteremic urinary tract infection (*17*).

In previous studies, some *E. coli* O78:H^–^ strains have been linked with septicemia in calves and piglets and in avian extraintestinal infections, such as respiratory infections, colicepticemia, and cellulitis (*18**,**19*). They also have been isolated from humans with extraintestinal infections, such as urinary tract infections, sepsis, and meningitis (*20*). Diarrheagenic *E. coli* strains do not usually cause extraintestinal diseases. STEC O78:H^–^ strains isolated from human gut often are linked with mild diarrhea and asymptomatic infections in humans (*21**–**25*). No invasive strains of serotype O78:H^–^ have been detected (Flemming Scheuz, pers. comm.).

In STEC infections, the Stx variant produced by the strain is commonly the main risk factor for development of HUS. However, the toxin itself might not be sufficient to cause HUS; other bacterial and patient factors also play a role. The isolated strains produced the toxin variant 1c, which has been linked with mild clinical signs or with asymptomatic carriage (*5**,**23*). A prominent feature of STEC carrying *stx_1c_*, which was also found here, is lack of the *eaeA* gene encoding intimin, suggesting the absence of the locus of enterocyte effacement (*23*). On the basis of these findings, the invasive STEC described in this study is likely to have a variety of other still unknown critical virulence factors that affect its pathogenesis and its ability to spread into the bloodstream.

HUS develops in ≈5%–15% of patients <10 years of age in whom *E. coli* O157:H7 infection is diagnosed and occurs 2–14 days after diarrhea onset (*8*). In contrast to the O157-related HUS cases, less information is available about the non–O157-related HUS cases. Some risk factors, including an elevated leukocyte count, administration of antimicrobial drugs, use of antimotility agents, and very young age, are associated with increased risk for HUS (*8*). No specific therapy exists for STEC infections, but antimicrobial drugs, antimotility agents, opioids, and nonsteroidal anti-inflammatory drugs should not be given to acutely infected patients (*8**,**26*). On the other hand, for infants or immunodepressed patients with enteritis, particularly when bacteremia is suspected, antimicrobial drug therapy is fundamental to controlling the disease. Here, ceftriaxone, a third-generation cephalosporin, was given to the neonate. Asymptomatic carriages in some patients, mainly adults, over a 1-year period have been reported (*27*). In this study, the neonate’s mother shed STEC bacteria at 21 days, his father at 141 days, his sister at 122 days, and the neonate at 117 days (there are no data regarding shedding for his brother). To prevent further bacterial shedding, probiotics such as *Lactobacillus* spp. and *Saccharomyces boulardi* were given after 1 month’s carriage to the father and the siblings, but they had no effect on eliminating carriage.

Ruminants, such as cattle and sheep, are the major reservoir of STEC (*28*). None of the family members, however, had contact with any farm animals, and the family had no pets. One of the family members of the neonate might have been infected with STEC by eating contaminated food, but these food items were not available for investigation. Moreover, because all the family members were asymptomatic, estimating the exact date of their infections is difficult. Secondary infections among family members most likely resulted from person-to-person transmission or from food given to the children with contaminated hands of other family members or from some other cross-contamination. Family clusters have been reported to be common (*29*). In Finland, ≈50% of STEC infections are family related (*30*).

Handwashing practices may be of greater relevance than food as a source of infection in infants and very young children because the infection might result from an infected person or animal in the home. Prolonged excretion of STEC and intimate caring of infants by family members provide a risk for cross-infections. Therefore, to limit the risk for STEC infection, thorough handwashing before touching food or young babies is particularly necessary.

## References

[1] Karmali MA, Petric M, Lim C, Fleming PC, Arbus GS, Lior H. The association between idiopathic hemolytic uremic syndrome and infection by verotoxin-producing *Escherichia coli.* J Infect Dis. 1985;151:775–82. 10.1093/infdis/151.5.775 3886804

[2] Cantey JR, Moseley SL. HeLa cell adherence, actin aggregation, and invasion by nonenteropathogenic *Escherichia coli* possessing the *eae* gene. Infect Immun. 1991;59:3924–9. 1682254 10.1128/iai.59.11.3924-3929.1991 PMC258978

[3] Luck SN, Badea L, Bennett-Wood V, Robins-Browne R, Hartland EL. Contribution of FliC to epithelial cell invasion by enterohemorrhagic *Escherichia coli* O113:H21. Infect Immun. 2006;74:6999–7004. 10.1128/IAI.00435-06 16982828 PMC1698073

[4] Herold S, Karch H, Schmidt H. Shiga toxin–encoding bacteriophages—genomes in motion. Int J Med Microbiol. 2004;294:115–21. 10.1016/j.ijmm.2004.06.023 15493821

[5] Zhang W, Bielaszewska M, Kuczius T, Karch H. Identification, characterization, and distribution of a Shiga toxin 1 gene variant (stx(1c)) in *Escherichia coli* strains isolated from humans. J Clin Microbiol. 2002;40:1441–6. 10.1128/JCM.40.4.1441-1446.2002 11923370 PMC140390

[6] Kaper JB, Nataro JP, Mobley HL. Pathogenic *Escherichia coli.* Nat Rev Microbiol. 2004;2:123–40. 10.1038/nrmicro818 15040260

[7] Karmali MA. Host and pathogen determinants of verocytotoxin-producing *Escherichia coli*–associated hemolytic uremic syndrome. Kidney Int Suppl. 2009; (112):S4–7. 10.1038/ki.2008.608 19180132

[8] Tarr PI, Gordon CA, Chandler WL. Shiga-toxin–producing *Escherichia coli* and haemolytic uraemic syndrome. Lancet. 2005;365:1073–86. 10.1016/S0140-6736(05)71144-2 15781103

[9] Karch H, Tarr PI, Bielaszewska M. Enterohaemorrhagic *Escherichia coli* in human medicine. Int J Med Microbiol. 2005;295:405–18. 10.1016/j.ijmm.2005.06.009 16238016

[10] Clinical and Laboratory Standards Institute. Methods for dilution antimicrobial susceptibility tests for bacteria that grow aerobically. Approved standard—eighth edition. Wayne (PA): The Institute; 2009 [cited 2011 Aug 1]. http://www.clsi.org/source/orders/free/m07-a8.pdf

[11] Ratiner YA. Serotyping of *Escherichia coli* flagellar antigens [in German]. In: Stein G, Fünfstück R, editors. Harnwegsinfektionen. Aktuelle Gesichtspunkte zur Pathogenese, Diagnostik und Therapie. II Wissenschaftliches Symposium, Jena, 1989 Aug 30–Sep 1. Frankfurt am Main: pmi-Verlag GmbH; 1991. p. 47–51.

[12] Antikainen J, Tarkka E, Haukka K, Siitonen A, Vaara M, Kirveskari J. New 16-plex PCR method for rapid detection of diarrheagenic *Escherichia coli* directly from stool samples. [PubMed ]. Eur J Clin Microbiol Infect Dis. 2009;28:899–908. 10.1007/s10096-009-0720-x 19238467

[13] Baldini MM, Kaper JB, Levine MM, Candy DC, Moon HW. Plasmid-mediated adhesion in enteropathogenic *Escherichia coli.* J Pediatr Gastroenterol Nutr. 1983;2:534–8. 10.1097/00005176-198302030-00023 6352891

[14] Keskimäki M, Mattila L, Peltola H, Siitonen A. Prevalence of diarrheagenic *Escherichia coli* in Finns with or without diarrhea during a round-the-world trip. J Clin Microbiol. 2000;38:4425–9. 11101575 10.1128/jcm.38.12.4425-4429.2000 PMC87616

[15] PulseNet. One-day (24–48 h) standardized laboratory protocol for molecular subtyping of *Escherichia coli* O157:H7, non-typhoidal *Salmonella* serotypes, and *Shigella sonnei* by pulsed field gel electrophoresis (PFGE) [cited 2011 Aug 1]. http://www.cdc.gov/pulsenet/protocols/ecoli_salmonella_shigella_protocols.pdf

[16] Paton AW, Beutin L, Paton JC. Heterogeneity of the amino-acid sequences of *Escherichia coli* Shiga-like toxin type-I operons. Gene. 1995;153:71–4. 10.1016/0378-1119(94)00777-P 7883188

[17] Chiurchiu C, Firrincieli A, Santostefano M, Fusaroli M, Remuzzi G, Ruggenenti P. Adult nondiarrhea hemolytic uremic syndrome associated with Shiga toxin *Escherichia coli* O157:H7 bacteremia and urinary tract infection. Am J Kidney Dis. 2003;41:E4. 10.1053/ajkd.2003.50022 12500215

[18] Farooq S, Hussain I, Mir MA, Bhat MA, Wani SA. Isolation of atypical enteropathogenic *Escherichia coli* and Shiga toxin 1 and 2f-producing *Escherichia coli* from avian species in India. Lett Appl Microbiol. 2009;48:692–7. 19413811 10.1111/j.1472-765X.2009.02594.x

[19] Parreira VR, Gyles CL. Shiga toxin genes in avian Escherichia coli. Vet Microbiol. 2002;87:341–52. 10.1016/S0378-1135(02)00084-6 12069771

[20] Gophna U, Oelschlaeger TA, Hacker J, Ron EZ. Yersinia HPI in septicemic Escherichia coli strains isolated from diverse hosts. FEMS Microbiol Lett. 2001;196:57–60. 10.1111/j.1574-6968.2001.tb10540.x 11257548

[21] Beutin L, Zimmermann S, Gleier K. Human infections with Shiga toxin–producing Escherichia coli other than serogroup O157 in Germany. Emerg Infect Dis. 1998;4:635–9. 10.3201/eid0404.980415 9866741 PMC2640265

[22] Chérifi A, Contrepois M, Picard B, Goullet P, Orskov I, Orskov F. Clonal relationships among Escherichia coli serogroup O78 isolates from human and animal infections. J Clin Microbiol. 1994;32:1197–202. 8051245 10.1128/jcm.32.5.1197-1202.1994 PMC263643

[23] Friedrich AW, Borell J, Bielaszewska M, Fruth A, Tschäpe H, Karch H. Shiga toxin 1c–producing Escherichia coli strains: phenotypic and genetic characterization and association with human disease. J Clin Microbiol. 2003;41:2448–53. 10.1128/JCM.41.6.2448-2453.2003 12791863 PMC156474

[24] Shaheen HI, Khalil SB, Rao MR, Abu Elyazeed R, Wierzba TF, Peruski LF Jr, Phenotypic profiles of enterotoxigenic *Escherichia coli* associated with early childhood diarrhea in rural Egypt. J Clin Microbiol. 2004;42:5588–95. 10.1128/JCM.42.12.5588-5595.2004 15583286 PMC535259

[25] Stephan R, Ragettli S, Untermann F. Prevalence and characteristics of verotoxin-producing *Escherichia coli* (VTEC) in stool samples from asymptomatic human carriers working in the meat processing industry in Switzerland. J Appl Microbiol. 2000;88:335–41. 10.1046/j.1365-2672.2000.00965.x 10736003

[26] Cimolai N, Morrison BJ, Carter JE. Risk factors for the central nervous system manifestations of gastroenteritis-associated hemolytic-uremic syndrome. Pediatrics. 1992;90:616–21. 1408519

[27] Kuusi M, Eklund M, Siitonen A, Virkki M, Häkkinen P, Mäkelä R. Prolonged shedding of Shiga toxin–producing *Escherichia coli.* Pediatr Infect Dis J. 2007;26:279. 10.1097/01.inf.0000256733.22690.4d 17484238

[28] Pennington H. *Escherichia coli* O157. Lancet. 2010;376:1428–35. 10.1016/S0140-6736(10)60963-4 20971366

[29] Karch H, Bielaszewska M, Bitzan M, Schmidt H. Epidemiology and diagnosis of Shiga toxin–producing *Escherichia coli* infections. Diagn Microbiol Infect Dis. 1999;34:229–43. 10.1016/S0732-8893(99)00031-0 10403103

[30] Eklund M, Nuorti JP, Ruutu P, Siitonen A. Shigatoxigenic *Escherichia coli* (STEC) infections in Finland during 1998–2002: a population-based surveillance study. Epidemiol Infect. 2005;133:845–52. 10.1017/S0950268805004450 16181504 PMC2870315

